# Barriers and motivators of Ghanaian and African‐Surinamese migrants to donate blood

**DOI:** 10.1111/hsc.12692

**Published:** 2018-11-27

**Authors:** Elisabeth F. Klinkenberg, Elisabeth M. J. Huis in ’t Veld, Puck D. de Wit, Wim L. A. M. de Kort, Mirjam P. Fransen

**Affiliations:** ^1^ Donor Studies Sanquin Research Amsterdam The Netherlands; ^2^ Department of Public Health Amsterdam UMC, Amsterdam Public Health Research Institute, University of Amsterdam Amsterdam The Netherlands; ^3^ Department of Medical and Clinical Psychology Tilburg University Tilburg The Netherlands

**Keywords:** blood donors, Ethnic Minorities, motivation, qualitative research, Sub‐Saharan African, the Netherlands

## Abstract

African migrants are underrepresented as blood donors in many Western countries, which can lead to shortages of specific blood types for transfusion. More insight in the reasons for this underrepresentation is required to improve blood donor recruitment and retention strategies. The aim of this qualitative study was to explore barriers and motivators for donating blood among migrants of African background. The research population consisted of first and second generation African‐Surinamese (*n* = 20) and Ghanaian (*n* = 16) migrants living in the Netherlands. In semi‐structured personal interviews performed in 2016 and 2017, their experiences and opinions regarding blood donation, barriers, and motivators to (not) become a blood donor and their suggestions to improve recruitment were explored. Data collection was continued until data saturation was achieved. The interviews revealed that although all participants knew about blood donation in general, only four had previously heard of the Dutch national blood bank organisation. Participants expected that if blood was needed, the blood bank would directly approach them, as in their country of origin. Other main blood donation barriers were fear (e.g., of needles, losing too much blood) and issues related to health and non‐eligibility to donate. Main motivators were mainly of altruistic nature (e.g., saving a life) and an increased awareness of the need via personal recruitment appeals. It is concluded that expectations regarding donor recruitment—derived from the country of origin—and unawareness of the need for blood can act as important barriers in blood donation among African migrants. Contrary to studies in the United States and Australia, perceived discrimination and social exclusion did not seem to be a donation deterrent among migrants in the Netherlands. Creating awareness of the need of blood by actively approaching, and informing migrants about the donation procedure in the host country, should be considered by blood banks.

**Abbreviations**
BCA: Blood Collection Agencies
SSAs: Sub‐Saharan Africans

**What is known about this topic**

- African donors are underrepresented in high‐income Western countries, while their donations are important for precise blood‐matching in chronic transfusion patients.
- Most studies on blood donation among African communities in high‐income countries are done among African‐Americans in the United States, while studies on the European context are lacking.

**What this paper adds**

- Expectations derived from experiences in the country of birth, play a large role in why African migrant communities did not contemplate about blood donation in their host country.
- Personal recruitment and awareness raising are needed to reach and inform African migrants.
- Unlike earlier studies, discrimination and social exclusion were not reported as major barriers in the Dutch context.

## 1 INTRODUCTION

The recruitment and retention of blood donors from various ethnic backgrounds is increasingly important for an adequate supply of blood. However, in many Western countries, migrant minorities are underrepresented in the blood donor pool (van Dongen, Mews, de Kort, & Wagenmans, 2016). This underrepresentation presents a problem, since ethnic diversity in Western society is growing and some minority groups differ in extended blood typing compared to a country's majority population (Reid, Lomas‐Francis, & Olsson, 2002). One important group with unique blood phenotypes, as compared to people of Western‐European origin, are people from Sub‐Saharan African (SSA) origin (Howes et al., 2011). When the blood types of donor and patient cannot be fully matched, the patient may become alloimmunised and future incompatible transfusions may lead to fever, hypotension, and even death. To prevent alloimmunisation and its serious complications, SSA patients are often in need of precisely matched blood, also with regard to these extended blood types (Yazdanbakhsh, Ware, & Noizat‐Pirenne, 2012). Although migrant SSAs are underrepresented as donors, SSAs are not underrepresented as chronic transfusion patients (Miller et al., 2013). To ensure a sufficiently diverse and stable blood supply, more SSAs need to be recruited and retained as blood donors.

Previous studies on blood donation barriers and motivators among African minorities and migrant groups, demonstrated that discrimination, social exclusion, and distrust are often perceived and experienced as barriers for donating blood, as they felt their blood would not be wanted by the white majority population and be wasted (Polonsky, Brijnath, & Renzaho, 2011; Tran, Charbonneau, & Valderrama‐Benitez, 2013). Also, preferences for donating within the own community or family, and symbolism surrounding blood are indicated in various studies (Charbonneau & Tran, 2013; Grassineau et al., 2007). But a systematic review on African donation barriers and facilitators also demonstrated that general factors play an important role, such as fear, convenience, and eligibility (Glynn et al., 2006; Klinkenberg et al., 2018).

Most studies are focused on African‐Americans in the United States, with a few performed on African communities in Canada and Australia (Charbonneau & Tran, 2013; Polonsky, Renzaho, & Brijnath, 2011; Schreiber et al., 2006; Shaz, Demmons, Hillyer, Jones, & Hillyer, 2009). Perspectives from European countries are lacking. While the challenges and needs of recruiting more African minorities and migrants are similar worldwide, the socioeconomic contexts are not (Kivisto, 2002). Most African minorities in the United States are living there for many generations, while in Europe, most African minorities migrated from Africa to another country, or are born from migrants. These different upbringings and contact with the country of origin, may bear on different perceptions and experiences with blood donation compared with the native majority group or even minority groups who are settled in a country for multiple generations. Therefore, more insights in specific African migrant communities in Europe are needed to improve blood donor recruitment strategies.

### 1.1 The Dutch context

The Dutch national blood collection agency (BCA), named Sanquin, depends on voluntary, non‐remunerated blood donors who can donate up to three to five times each year and is the only BCA responsible for the Dutch blood supply (Sanquin, 2016). As in many other countries, migrant recruitment and blood‐matching is becoming an important subject for research and practice because of growing migration and thus the growing demand of specific blood products (van Dongen et al., 2016). In 2017, more than 240 thousand SSAs live in the Netherlands, representing about 1.4% of the total Dutch population (Statistics Netherlands, 2016). A relatively large part of the SSAs originates from Ghana (23,000; 11.2%). However, the largest group of African descendants (not from the African mainland) originates from Surinam. This group is often referred to as African‐Surinamese or Creoles, and about 39,000 of this Surinamese sub‐group were estimated to live in the Netherlands in 2008, for which unfortunately no new statistics are known (Oudhoef, Harmsen, Loozen, & Choenn, 2011). Although the number of SSA descendants—both from former colonies and the African mainland—is relatively small compared to the Turkish (397,000) and Moroccan (386,000) communities, the SSA community is expected to grow and differ more on extended blood typing (Carling & Hernández‐Carretero, 2011; Connell, Zurn, Stilwell, Awases, & Braichet, 2007; Statistics Netherlands, 2015). The majority of both groups live in the capital of the Netherlands: Amsterdam.

To explore the blood donation barriers and motivators of these two large African groups, and find more efficient ways to recruit and retain them as blood donors, the PRECEDE‐PROCEED model was selected to guide our assessment (Green & Kreuter, 1991). This framework can assist in designing efficient health programs. The first two phases are concerned with the desired outcomes, namely increasing blood donors from SSA communities, decreasing alloimmunisation, and increasing the quality of life of SSA transfusion patients. Barriers and motivators to donate blood can, if modified, result in behaviour change and these desired outcomes. These can be divided into predisposing, reinforcing and enabling factors. Predisposing factors are defined as knowledge, attitudes, beliefs, values, and perceptions that facilitate or hinder motivation to change. Reinforcing factors are defined as rewards and/or punishments that facilitate or hinder the desired behaviour. Enabling factors are defined as skills, health, and resources that can facilitate or hinder these desired behavioural changes, as well as environmental changes. This approach is part of a systematic intervention development using the Intervention Mapping protocol (Bartholomew, Parcel, Kok, Gottlieb, & Fernandez, 2011), in which the results from multiple studies form the basis of new SSA blood donor recruitment and retention strategies. This particular study aimed to identify barriers and motivators experienced by African‐Surinamese and Ghanaian migrants to become a blood donor in the Netherlands, with the aid of this framework.

## 2 MATERIALS AND METHODS

### 2.1 Participants and recruitment

Individuals for semi‐structured qualitative interviews with an African‐Surinamese background were recruited from participants in the Healthy Life in an Urban Setting (HELIUS) study in Amsterdam (Snijder et al., 2017; Stronks et al., 2013). The HELIUS study is a prospective cohort study among a multi‐ethnic population, with a main focus on cardiovascular diseases, infectious diseases, and mental health. The African‐Surinamese participants of the HELIUS study were personally asked to participate in the interview after the main HELIUS examination. Ghanaian participants were recruited via a certified care provider, who focused mainly on the care of African communities.

People were personally invited to participate in an interview if at least one parent was born in Ghana or Surinam, or if the participant was born in that country himself/herself and was aged 18 years or older. Blood donor status and eligibility to donate were not inclusion criteria for this interview study. Participants had to be fairly fluent in Dutch or English.

A total of 36 interviews were completed: 20 with African‐Surinamese and 16 with Ghanaian participants (Table 1). The interviews lasted on average 30 min. In both groups, more women than men participated. Three persons were second‐generation migrants. None of the participants was currently a blood donor, whereas about 25% of the participants had donated blood in the past. From those who donated in the past, three participants reported to have donated in the Netherlands, while six had only donated in their country of birth.

**Table 1:** Characteristics of the interviewed participants (*n* = 36)

	African‐Surinamese		Ghanaian		Total	
	*n*	%/*M* (*SD*)	*n*	%/*M* (*SD*)	*n*	%/*M* (*SD*)
Gender						
Men	5	25	6	37.50	11	30.60
Women	15	75	10	62.50	25	69.40
Age in years						
18–35	4	20	4	25	8	22.22
36–55	9	45	6	37.50	15	41.67
55+	7	35	6	37.50	13	36.11
Years in the Netherlands	18	34.44 (12.04)	16	22.88 (11.64)	34	29 (13.22)
Generation						
First	18	90	15	93.75	33	91.70
Second	2	10	1	6.25	3	8.30
Donor status						
Never donor	16	80	11	68.75	27	75
Ever donor	4	20	5	31.25	9	25
Current donor	0	0	0	0	0	0
Total	20		16		36	

### 2.2 Data collection

Semi‐structured, personal interviews were performed in 2015 and 2016 by two authors (PW and EK) using one interview guide (Appendix S1). Both interviewers were female, of Dutch background and trained in qualitative research methods with prior interview experience. Interviews were performed in Dutch or English, depending on the participants’ preference. All interviews were conducted in Amsterdam, in a relatively private setting such as a separate office. Interviews were continued till data saturation was achieved when no new barriers or motivators for blood donation were attained compared to the earlier done interviews, guided by the theoretical PRECEDE‐PROCEED model (Aldiabat & Le Navenec, 2018; Fusch & Ness, 2015; Green & Kreuter, 1991).

The interview guide was developed by authors MF, PW, and EK based on the main determinants distinguished in the literature on African minority or migrant blood donation barriers and motivators (Klinkenberg et al., 2018). These determinants were: knowledge, awareness, fear, health, deferral and exclusion factors, attitudes, religion, mistrust, ethnic identification, discrimination, social exclusion, (in)convenience, mistrust, altruism, incentives, and health checks. Before each interview, background information about the goal and content of the interview was provided, and all participants were informed that the interview was anonymous, voluntary and that the participant could choose to withdraw from the interview at any given time without giving a reason. Participants were asked to give oral consent for research participation and recording of the interview before and after the tape‐recording started. None of the participants declined the interview after explanation of the goals and all gave oral consent. At the end of the interview, the interviewer repeated the general answers, asked the participant whether this was correct and whether the participant had more to add. Additional questions were added to the interview guide based on new knowledge from former interviews. The project protocol this study is part of was reviewed by the Medical Ethics Review Committee of the Academic Medical Center Amsterdam. This study was waived from requiring medical ethical approval, because this protocol does not fall under the Medical Research Involving Human Subjects Act of the Dutch law.

### 2.3 Data analysis

**Figure 1:**
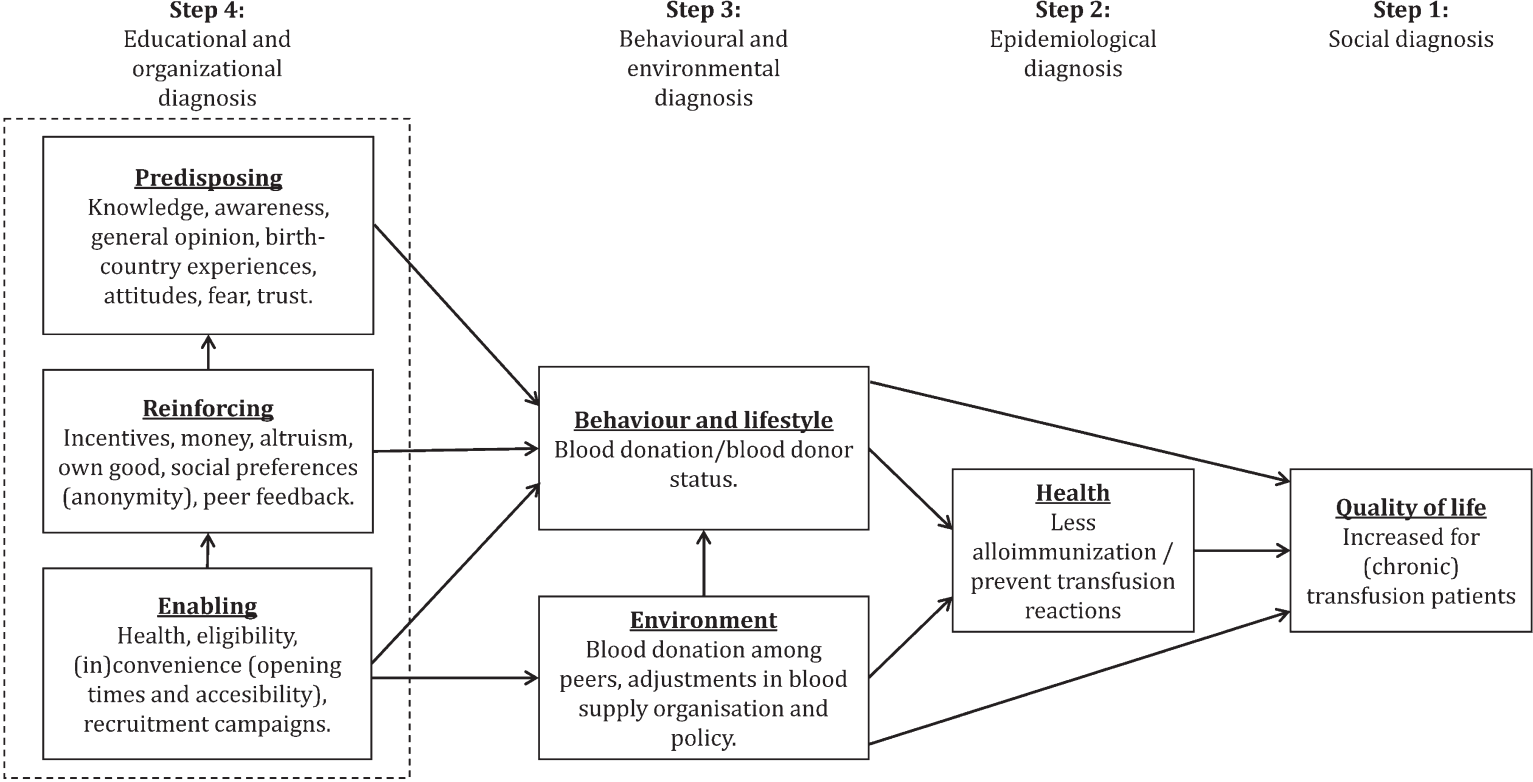
The PRECEDE‐PROCEED model based on blood donation among African migrants, with identified preceding, reinforcing and enabling factors in Step 4

All interviews were recorded and transcribed completely. The transcripts were coded and analysed in MAXQDA software for qualitative data analysis (version 12, VERBI, GmbH, Germany). Thematic analysis was done by authors MF and EK. Based on the PRECEDE‐PROCEED model, a coding framework was developed in which the barriers/motivators were divided into predisposing, reinforcing, and enabling factors (Figure 1) (Green & Kreuter, 1991). For the analysis, we focussed on step 4 of the model.

During the analysis, the researchers could extend the coding framework if a theme emerged from the data that was missing from the initial framework. After two transcripts were coded, the authors compared their individual analysis and resolved differences. When intercoder conformity was reached and no new themes were identified, author EK coded the remaining transcripts and re‐read all transcripts to ensure that they were coded appropriately. A professional translator translated the quotations from Dutch interviews to improve the quality of the transference of the findings (van Nes, Abma, Jonsson, & Deeg, 2010).

## 3 RESULTS

### 3.1 Predisposing factors

#### 3.1.1 Awareness and knowledge

Of the 36 participants, only four had heard of the Dutch national BCA (11%). The name (Sanquin) was often not known or not recognised, as one participant noted: ‘Where it is, what it is? […] if you say the name I wouldn't have any idea what you're talking about.’ (Female, 60+ years, Surinamese background). Although basic knowledge was present—as they knew what blood donation is and knew there are different types of blood—most participants explained that they did not know much about the procedure of blood donation in the Netherlands, such as where to donate. The lack of awareness seemed associated with never having heard, thought, talked about, or been approached regarding blood donation in the Netherlands, which is illustrated by a participant who had previously donated in Ghana: “It's new to me, that I am hearing today that you are doing research about blood. But I have not heard of any organisation or any group asking people to come forward to donate blood since I came to Europe.” (Male, 64 years, Ghanaian background).

The participants expected to be approached when blood was needed, which was often the case in their country of birth. They were generally more aware and knowledgeable of blood donation in their country of origin, even when they lived there for a shorter amount of time than in the Netherlands. For instance, participants from Ghana told that people are often requested to donate blood at organisations or in schools when the blood supply is running low: ‘In Ghana I had done it in school once. […] A few doctors came by and told us what we were going to do. I thought it was fun to do and I found it sad for the people who needed it, so I thought: why not?’ (Female, 22 years old, Ghanaian background).

#### 3.1.2 General opinion and attitudes

Thirty‐three participants had a positive opinion about blood donation (92%). It was generally considered a good thing to do and they mentioned that they would encourage people to do it. A Surinamese man said: “I think it's really good that people are willing to do that sort of thing. I'm very happy about that.” (around 55 years) and a Ghanaian man told: “Blood donation is good, because it can sustain peoples” lives ‐ my life, your life, the life of my children, and other children's lives also.’ (53 years). But although the general opinion about blood donation was positive for most Surinamese and Ghanaian participants, 20 participants reacted hesitantly when asked if they would donate themselves (56%): “It's good that blood is being donated, but I don't know if I would do it myself.” (Female, 60+ years, Surinamese background).

#### 3.1.3 Religious attitudes and symbolism of blood

Although all participants had a Christian background, the denominations varied. For most participants identifying themselves as Catholic or Protestant, the religion had no direct link with barriers/motivators for blood donation. However, for Jehovah's Witnesses, blood transfusions and blood donations are prohibited because the Bible states that one should not “eat” blood. Two Ghanaian Jehovah's Witnesses said they would not donate blood because of their religion. One Ghanaian told his wife lost too much blood during labour, but was not allowed to receive blood: “Fortunately, there was the church member who was a doctor, so he rescued her so that she shouldn't eat the blood. She must eat something like vegetables, fruit and other. And within a week time she got a bit better.” (61 years old).

For 26 participants, blood had a symbolic value (72%). Some of these symbolic meanings (particularly for Ghanaians) were closely connected to Christianity: “He [Jesus] sacrificed his life for me and—thanks to his blood—I'm a new person. Thanks to his blood I can say I'm Christian, and it allows me to go to Heaven. Blood has a kind of connection with him—a kind of bond between Jesus and me.” (Female, 22 years old). Blood as representing life was the most common symbolic relation, followed by the idea that blood represents health and human functioning.

#### 3.1.4 Fear and mistrust

Fear was reported by 15 participants (42%)—mainly females—as a donation barrier. Although the types of fear varied, the fear of seeing blood was commonly reported: “I'm terribly frantic about blood—I can't stand it. If I see only one drop when I've cut myself, then I just faint.” (Female, around 45 years, Surinamese background). Fear of needles and pain was also mentioned by several. Participants who did not report fear themselves, often said that fear was an important barrier in their community: ‘In the Surinamese community the obstacle is fear. […] We Surinamese often think that—if we donate—then there's not enough left for ourselves.’ (Female, 55 + years, Surinamese background).

Also, uncertainties and suspicions about what happens with the blood were mentioned. One Surinamese woman explained as follows: ‘I have blood that could be given […] but I'm cautious. I have to have a good feeling about it. Of course, if they'll say it will be of good use, but do they really use it that way? […] You won't find out if they use it for other purposes.’ (37 years. Although some participants expressed these uncertainties, the Dutch national BCA was not mistrusted despite most were not familiar with it: “I trust it very well, because of the machines and computer. I trust it a hundred percent.” (Female, 46 years, Ghanaian background). “I don't think they are joking with the safety of blood. I think everything goes well; in the Netherlands everything is done right.” (Female, 27 years old, Surinamese background).

### 3.2 Reinforcing factors

#### 3.2.1 Altruism

All participants mentioned that an important reason to donate blood was based on altruism, that is unselfish devotion to others’ well‐being. Participants argued that you should not expect something in return for doing a good deed such as donating blood and some participants seemed to compare donating blood with voluntary work or giving a gift to someone: “Voluntary. You give to give. From your hearts.” (Female, 46 years, Ghanaian background). Also, giving blood was often seen as a moral responsibility and as compassion towards the patient: ‘The child needs blood to survive and if somebody donates to this child, he might become the next president or a pilot. […] If that person will not get blood, he will die and everything is gone. So blood donation is good […] Just from the hearts and own will.’ (Male, 53 years old, Ghanaian background).

Although it was not as often reported as purely altruistic motivators, seven participants mentioned immaterially benefiting from donating blood would motivate them (19%). One example is experiencing positive emotional feelings, as a participant explained who had donated previously: “It's just a nice feeling you get. You always get something back if you do something good, if you help others. Really.” (Female, 52 years, Surinamese background).

#### 3.2.2 Incentives

Incentives, such as money and gifts, were generally not considered as motivating: ‘No, that [receiving something in return] isn't necessary […] Some people might say that if they give, they want something in return. But I believe that most people don't think that way.’ (Female, 60+ years, Surinamese background). Several participants also mentioned that receiving money for donating blood would have negative consequences: “I don't think it is okay that you just go to the hospital and say: ‘I want to sell blood’ and they took it away from you. It's not healthy to me.” (Female, 49 years, Ghanaian background).

Although money and gifts were often not perceived as motivating, nine participants had a positive opinion about a small reimbursement in the form of food/snacks (25%). Receiving food from the BCA was often seen as a way to recover after giving blood: “Maybe cake, apple, vegetables, broccoli, and chocolate drinks in a small handbag. After donating voluntary, they give you a bag like this as a present to you.” (Male, 64 years, Ghanaian background). “Tea, chocolate milk ‐ definitely chocolate milk. We Surinamese people have the idea that cacao helps to recover your blood.” (Female, 55+ years, Surinamese background).

#### 3.2.3 Anonymity, family donation preferences, and peer influence

In the Netherlands, donors do not know the recipient and vice versa. Two of the four Surinamese past donors, and one of the five Ghanaian past donors, mentioned they had given blood specifically for a family member. However, when asked if they were interested in who received their donated blood, most participants reacted indifferent and thought that this should be irrelevant: “If someone needs the blood, he just needs it, no matter who it gives. And the other way around too; if I need blood it won't matter to me from who it is, as long it is good and healthy blood.” (Male, 28 years, Surinamese background).

Four participants mentioned preferences regarding the recipient of their blood donation (11%); this was mainly observed among Surinamese woman with children. The preference often goes together with some kind of fear, for example for needles, or your blood going into someone else's body (i.e., seen as giving a part of yourself to a stranger): “I need to help my child. It may be unfair because it's my own child, but my blood is in there, so it doesn't matter. But for a stranger? I don't know.” (Female, 60+ years, Surinamese background).

Although for some participants family is imperative as the recipient of a blood donation, their opinions on blood donation were generally seen as unimportant. The participants perceived blood donation as an individual choice: ‘Look, in the Netherlands you learn to make your own choices. So, in the end, it's my choice if I want to do it or not. I“m old enough.” (Female, 30 years, Ghanaian background). However there is evidence that when close family members donate blood, the participant is more inclined to give blood: “Yes, their [my family members”] experiences were very good. In Surinam they did it and here they also do it. That's why I also wanted to do it.’ (Female, 60+ years, Surinamese background).

### 3.3 Enabling factors

#### 3.3.1 Health

Some participants mentioned that they did not donate blood due to their current health status. One Ghanaian man who had donated in the past said that he was not able to donate blood anymore: “I would donate it! But, this one [points to right arm] stroke. From my hand and leg” (62 years). Also, one Ghanaian woman said that she wanted to become a donor, but that it was not possible for her: “I gave one time and then a doctor told me my blood is not good because of my hepatitis B.” (46 years). Five Surinamese respondents mentioned a low haemoglobin level or iron deficiency as a barrier (25%). Based on our observations and the participants’ reports, it is estimated that at least five of the Ghanaian participants (31%) and four of the Surinamese participants (20%) would be definitely excluded from blood donation with the current health eligibility criteria of the Dutch BCA, for instance, due to earlier transfusions or reported infectious diseases.

#### 3.3.2 Practicalities

In general, participants felt they should be asked to donate at a convenient moment; otherwise they will not do it: “I think the only thing is the circumstance ‐ if they ask if I can come on this or that day and I'm not able to at that moment.” (Male, 60+ years, Surinamese background). It was also noted by most participants that the donation site should be in their own neighbourhood: ‘… if it was around the corner from my house. So, if it was easy, and they'd say: “come along to donate blood”, then I would do it.’ (Male, 28 years, Surinamese background). However, time constraints and accessibility of the donation site did not seem to be a major barrier for the participants if the need for blood was high.

#### 3.3.3 Recruitment strategies

The participants were asked how the recruitment of the BCA might be improved, and what kind of strategy would motivate them. One desired strategy was to make people more aware and inform them about the BCA and blood donation: ‘They could go just into the streets, to a market. But also advertise in the newspapers and do commercials on TV and radio […] they can give more information about this because a lot of people don't know what blood donation is all about … and you're doing it right now, with this. So, if I don't know anything about blood donation, you can explain everything to me.’ (Female, 24 years, Ghanaian background).

Suggestions as to what types of resources could achieve more awareness and knowledge about blood donation were diverse: most often mentioned were folders, the regular media, and social media. However, the type of recruitment that produced the most positive responses was a personal approach: ‘Personal! […] A folder ‐ well, I just put that aside … but if you go to people's homes and tell them about it, then they understand it better.’ (Female, 60+ years, Surinamese background). Surinamese participants mentioned festivals or football matches and Ghanaians mentioned communities or churches in particular as suitable places to recruit people from their ethnic group. Also “confronting” people with the consequences if no blood is available, was mentioned by six participants: “A little bit scary. Like a child who had an accident. A catchy text with it, so that people get woken up.” (Man, 60+ years, Surinamese background).

Lastly, when asked if the participants had a specific role‐model in mind for recruiting African donors, the responses were two‐sided. On the one hand, participants mentioned that a recruiter from the own community would be most efficient: “If you want to reach a certain culture, it would definitely help if you let people from that culture recruit themselves.” (Male, 28 years old, Surinamese background). On the other hand, participants preferred someone with more expertise in the process of blood donation or its health effects: ‘That would be my doctor. I would ask: if I donate blood, what would it cost me? What may I gain? […] Then my doctor can advise me what to do, eat or just go for it. If nothing is a problem, I will try it.” (Female, 49 years old, Ghanaian background).

## 4 DISCUSSION

In the present study, interviews were conducted with African‐Surinamese and Ghanaian individuals to determine barriers and motivators for blood donation in the Netherlands. The main predisposing barrier was a lack of awareness. Participants generally had more knowledge about blood donation in their country of birth. But also fears associated with donating blood were regularly mentioned, such as fears related to needles, fainting, or losing too much blood. Regarding the reinforcing factors, altruism appeared to be the main driving force to donate blood. However, some participants mentioned family donation preferences or incentives in the form of foods or snacks as reinforcing motivators. Of the enabling factors, participants preferred a personal approach highlighting the need for blood, which was reported as a more usual practice in the country of origin. Finally, health problems and non‐eligibility were reported as major deterrents for blood donation, which is often found to be a barrier among people of African origin in other countries as well (Cable et al., 2011; Grassineau et al., 2007).

Some of our findings contrast with the results of earlier studies on African minorities. Personal discrimination and social exclusion were previously found to be major factors for not donating blood, for instance, because people felt their blood is not wanted by the majority population (Polonsky, Brijnath, et al., 2011; Tran et al., 2013). In our study, these factors were not reported and not perceived as a barrier to donate blood. This result might be attributed to the high level of social tolerance in the Netherlands (Weldon, 2006). However, this might also be a non‐finding since both interviewers were of Dutch ethnic background, which may have acted as a barrier to freely speak about experiences related to discrimination or social exclusion (Adida, Ferree, Posner, & Robinson, 2016). Mistrust of the BCAs or the health system was also important barriers in other studies (Frye et al., 2014; Polonsky, Renzaho, et al., 2011). Although some of our participants reported they want more clarity about what happens with their blood, and a few reported negative experiences with Dutch health care, mistrust regarding donating blood or the BCA was not reported as a barrier.

A limitation is the inclusion of a highly specific, non‐randomly chosen group of participants. All were either from African‐Surinamese or Ghanaian descent, lived in Amsterdam and were fairly fluent in either Dutch or English. The identified factors in this group might differ as what would be found in a survey of a representative sample of this population. However, the qualitative character of this study aimed to reveal the types of specific barriers/motivators related to people of SSA descent, without quantitating them. Also, in the Netherlands, people are only eligible to donate blood if they can speak Dutch or English without the help of an interpreter. So from a blood donor recruitment perspective, it would have been unfeasible to include interview participants who are not fluent in Dutch or English. A strength of the study is that a diverse group was recruited in terms of age, past donor experiences and backgrounds, and the interviews were continued until saturation was achieved.

A major finding of this study is that practices from the country of origin, where more manifest blood donation appeals are made and people are directly asked to donate for a family member, influenced the expectations of blood donation in the Netherlands. People believed that the blood supply is sufficient, as they were never directly or indirectly asked to donate blood. The Dutch BCA rarely does appeal on family‐members of a transfusion patient and it is more common practice that potential‐donors register at the BCA individually on own initiative (World Health Organisation, 2017), which contrasts with the past donation experiences reported in the interviews, although Surinam now also depends entirely on voluntary, non‐remunerated donors. In Ghana, less than half donates voluntary and non‐remunerated. From the interviews, it can be concluded that these differences were not perceived as donation deterrents, but the participants were generally not aware of these country‐specific procedural differences. The expectations derived from the country of origin, should be taken into account in future research.

Since most participants were never reached by the Dutch BCA and had not thought about donating blood, it was sometimes difficult for them to consider other barriers. Yet, the overall opinion on blood donation was positive. Therefore, it is warranted to improve the visibility of the BCA. We do recognise, however, that building awareness alone is not enough to recruit and retain more donors, as multiple intervention studies have pointed out (Francis, Polonsky, Jones, & Renzaho, 2017; Frye et al., 2014). Therefore, other interventions based on the migrants’ expectations and desires should be examined, such as providing more clarity on what happens with the blood after donation and providing food packages. Still, the current eligibility criteria can act as a barrier once SSA minorities are recruited, as many participants reported health‐related issues that exclude them from blood donation. Besides, SSAs often travel to their country of birth and have relatively low haemoglobin levels (Cable et al., 2011; Grassineau et al., 2007). Communicating with this group about the necessity of the eligibility criteria to protect both the donor and patient, might increase successful donations as some deferral criteria are linked to lifestyle behaviour. Creating awareness of the need of blood by actively approaching migrants of African descent, and appropriately informing them about the donation procedures should be considered by BCAs aiming for a more diverse donor pool. A quantitative study will follow the current study, in which the identified barriers and motivators will be assessed among a large sample.

## CONFLICT OF INTEREST

None.
