# Supplementary material for: Barriers and motivators of Ghanaian and African‐Surinamese migrants to donate blood
Source: Health Soc Care Community. 2018 Nov 27;27(3):748–56. doi: 10.1111/hsc.12692 (PMC7379538; doi:10.1111/hsc.12692)
Supplement: Supplementary file 1 [file HSC-27-748-s001.docx]

**Appendix; interview guide**

**Introduction**Thank you for wanting to cooperate with this interview. Was it easy to come here?

Before we start I shall tell something about myself and about the procedure of this interview. I work as a researcher for the Academic Medical Center Amsterdam and Sanquin. We would like to know how people of Ghanaian and Afro-Surinamese descent think about blood donation, and why they do or do not want to donate.

This interview will take about half an hour to one hour of your time. Because I cannot remember everything of our conversation, this interview will be recorded. Your name and other private information will not be revealed in the study. Quotations and findings of this interview may be published, in scientific journals for example, but all information will be made anonymous.

The interview is voluntarily, so there are no consequences if you don’t want to give answers on certain questions, want to take a break from this interview, or want to quit the interview. You may stop any time and you don’t have to give a reason. Any information you have provided up to that point will be included in the study data, unless you ask me not to include it.

Because we would like to know exactly how people think about blood donation, I will ask many questions and will ask frequently to explain your views and experiences. There are no good or wrong answers. It is solely about your own opinion, views and personal experiences, and it is okay if you do not know how to answer or respond to certain questions.

Do you have any **questions** so far about the study or this interview? Is it okay if I start the **recorder** now?

***Starting questions:***Informed consent:
I just told you the **purpose and process** of this interview. Do you **understand these regulations** and do you wish **to continue with this interview**?

***Warming up:***
We will start out with some basic questions:
- What is your **age**?
- Are you **born in Surinam/Ghana**? **How long** do you live here **in the Netherlands**?
- Do you have **family living here** in the Netherlands?
- What are your **experiences with living in the Netherlands**?
- Can you tell me a little bit more about your **occupation**? What/where have you **studied**? What are your **interests**?

***Transition:***
We talked a little bit about the subject: blood donation
- Have you ever **heard of blood donation**? Do you know where you have heard this?
- Can you explain in your own words **what blood donation is**? How do you know this?
- Have you ever **donated blood yourself** in the Netherlands or another country? Do you **know others** who donated blood?

***Core questions:*
*Blood donation experiences: barriers, fear, deferral, attitude:***1. Have you ever **thought about donating** blood (again)?
- What are the most **important reasons** that you have (not) thought about donating blood?
- *if no answer can be given:* If you think about **other people**, what could be a reason for them to (not) donate?

2. How do you **feel about** blood donation?
- Would that **be a reason** to donate/not donate blood for you?
- - Did you ever **discuss blood donation** with someone else?

3. Did you ever **receive blood** yourself? If so, how did you feel about that/experienced this?
- Do you **know others** who received blood?

***Fear, health-related issues, deferral and knowledge***
4. Are you aware of **possible side effects** of donating blood?
- Yes: what kind of side effects? What do you think about that? Why? Is that a barrier for you to not donate blood?
- No: There are some people who can feel dizzy or tired, but in a few cases people can faint. What do you think about that? Why? Is that a barrier for you to not donate blood?
- Do you think there is a chance of getting infectious diseases through giving or taking blood?

5. Some people are **not allowed to donate**, due to **sickness** or a **risky lifestyle behaviour**. How do you feel about that? Can you explain more? What sickness or lifestyle behavior may hinder blood donation? Do you know if you are allowed to donate or not? Could that be a reason for you to not donate blood?

***Dutch blood bank and Dutch health care: knowledge, trust, safety***
6. What do you know about **blood donation in the Netherlands**?
- What do you think about the blood bank/**Sanquin**? Why? Have you ever heard of it? *show logo*
- Do you know where Sanquin is **located**? If so, how do you know?
- Do you know **what happens with the blood** when it is donated? Do you know what it is used for? If so, can you name some examples?
- Blood is tested on **infectious diseases**. How do you feel about that? If you would be a blood donor, would you want to know if you have infectious diseases or not?
- How much **trust** do you have in the way blood is given in the Dutch blood bank Sanquin? Can you tell me more about it?
- What do you think about the **safety** regarding giving or receiving blood? Do you think this is a reason for (not) donating?

7. What is your opinion about the **healthcare system** in the Netherlands?
- Do you **trust** the healthcare here?
- What are your **experiences with healthcare**?
- Do you think that your experiences in healthcare **plays a role in your view** towards the blood bank? Would it be a reason for you not to donate blood?

***Blood donation country of origin: barriers/facilitators, ethnic identification***
8. Do you know something about **blood donation in your country of birth**? Can you tell me something about that?
- Do you know how the blood bank in **Surinam/Ghana recruited blood donors**? If so, can you tell me something about that?
- What **reasons/motives** do people have there to donate?
- Do you have the idea that people **in Surinam/Ghana** are **more likely to donate** than Surinamese/Ghanaian people here **in the Netherlands**? If so, why would that be?
- Do you know if people can **give blood during their holiday** in their country of origin? If so, do your family members or acquaintances makes use of that?

***Family and acquaintances: kinship***
9. What do you think about what your **family and acquaintances know** about blood donation?
- What does **blood mean to them**? What do they think about regarding blood?
- Did your **family or acquaintances ever given blood** or are they blood donor right now? What is your opinion about that?
- Does **family or acquaintances ever received blood**, for example during an operation in the hospital. If so, how did they experience it?
- Would you **give blood if your family found it important**? Or wouldn’t you give blood if they found it a bad idea?

***Ethnicity and anonymity: kinship, trust, ethnic identification/discrimination***
10. Would **it matter** to you, if you would donate blood, **who receives your blood**? Who would you prefer to (not) receive your blood? Why (not)? Would you be more prepared to donate if you knew that somebody from the same ethnic background received your blood?

11. If you donate blood**, you won’t know who receives your blood**. What is your opinion about that? Why do you see it that way?
- If you ever need blood yourself, would **it matter** from **who this blood originates**?

12. Did you know there is a **shortage of blood**? How do you think this **shortage emerged**?
- Do you think it **is easier to donate blood for some people** compared to others? (if not well understood: are some groups probably more often excluded to donate blood than others?)

13. Would you give blood if a **family member or a good friend needs it**? Why (not)?
- Would people be **more likely to give blood** if they knew it would go to people from their **same background**? Why (not)?

***Convenience: time and place***14. If you were a blood donor, what would be a **convenient place** to donate? Why there? Would that also be convenient for other people? Do you think this is a reason for (not) donating?

15. If you were a blood donor, what would be a **convenient time** to donate? Why that time? Would that also be convenient for other people? Do you think this is a reason for (not) donating?

***Pro-social behavior: altruism vs. rewards***
16. Have you ever done **voluntary work**? If so, can you tell me more about it?
- What is your opinion about the fact that **donating blood is completely voluntary**?
- Do you think that **healthy people should give blood** to the people who are **not healthy**? Why (not)?
- In some countries, blood donors do get **rewards/money** for donating blood. How do you feel about that?

16. Some people donate blood solely for the purpose of **doing something good for others or society** in general, what do you think about that?

18. How do you feel about it that some people care for others **without getting anything in return**? Even when there is no real reward, what **would motivate** those people? Did you have **similar experiences**? Can you tell more about it?

***Blood donation in relation to religion***
19. Are you **religious**? If so, what is your belief?
- What is the **perception of your belief** (*of: religious people in general*) towards blood donation? Can you tell more about that?
- Is (your) **religion a reason to (not) donate blood**? Can you explain?

20. What does **blood mean to you**?
- Do you have a specific **symbolic value** regarding blood? Do you associate blood with a certain **concept or feeling**? If so, can you tell me more about it? 🡪 if the question is not well understood: Do you associate blood with a specific concept or feeling? Such as life, death, heritage, a biological substance…Does this perception make it harder/easier to donate blood?

***Blood donation tips for recruiting new donors***
21. Have you ever seen a **recruitment campaign of the Dutch blood bank**, for example a flyer? If so, what is your opinion about that? Was this flyer easy for you to understand? If not, why not? Which parts gave the most difficulties?

22. **What should the blood bank do to recruit** more donors?
- Why do you think that?
- What kind of **measurements could be most suitable** to be used for recruiting new donors?
- How can you **convince someone in your own environment** to donate blood?
- From **whom would you like to receive more information** regarding blood donation?
- What would you like to **know more about blood donation**? Do you think many people **lack knowledge** about blood donation? Why (not)?

23. Which **groups would be most motivated** to become blood donor you think? (e.g. men/women, young/old, first/second generation immigrants, educational level?) Why do you think that?
- What kind of person, **a role model**, would be most suitable for getting the message across **for that group**? When will this message have the most effect?

***Conclusion***We are almost at the end of our interview. If I have understood correctly, you told me that…is that correct? Which are the most important for you? Which reasons have the biggest impact on you? Are there other reasons for you which we haven’t talked about? Are there other things you would like to share?

Then I would like to thank you for your time. May we approach you in the future for follow-up studies?

After the recorder is stopped: how did you experience this interview? Did you feel comfortable talking about this subject? Did you have enough room to give your own views and opinions? Do you have recommendations for further interviews?
